# Potential protective roles of phytochemicals on glutamate-induced neurotoxicity: A review

**DOI:** 10.22038/ijbms.2020.43687.10259

**Published:** 2020-09

**Authors:** Amir R. Afshari, Sahar Fanoudi, Arezoo Rajabian, Hamid R. Sadeghnia, Hamid Mollazadeh, Azar Hosseini

**Affiliations:** 1 Department of Physiology and Pharmacology, School of Medicine, North Khorasan University of Medical Sciences, Bojnurd, Iran; 2 Department of Pharmacology, Faculty of Medicine, Mashhad University of Medical Sciences, Mashhad, Iran; 3 Pharmacological Research Center of Medicinal Plants, Mashhad University of Medical Sciences, Mashhad, Iran; 4 Division of Neurocognitive Sciences, Psychiatry and Behavioral Sciences Research Center, Mashhad University of Medical Sciences, Mashhad, Iran

**Keywords:** Excitotoxicity, Glutamate, Neurotransmitter, Neurodegenerative- disorders, Phytochemicals

## Abstract

Glutamate, as an essential neurotransmitter, has been thought to have different roles in the central nervous system (CNS), including nerve regeneration, synaptogenesis, and neurogenesis. Excessive glutamate causes an up-regulation of the multiple signaling pathways, including phosphoinositide-3 kinase/protein kinase B (PI3K/Akt), Akt/mammalian target of rapamycin (mTOR) protein, mitogen-activated protein kinase (MAPK)/extracellular signal-regulated kinase (ERK)1/2, and autophagy that are involved in neurodegenerative diseases pathophysiology. There are numerous findings on curcumin, astaxanthin, thymoquinone, and berberine, as natural products, which have outstanding effects in cell signaling far beyond their anti-oxidant activity, considering as a potential therapeutic target for glutamate excitotoxicity. Herein, we address the role of glutamate as a potential target in neurodegenerative diseases and discuss the protective effects of certain phytochemicals on glutamate-induced neurotoxicity.

## Introduction

Glutamate, as an excitatory neurotransmitter of central nervous system (CNS), plays a crucial role in memory, synaptic plasticity, learning, motor function, and neural transmission. Also, it has been proposed that glutamate can modulate nerve regeneration, tumor development, synaptogenesis, neurogenesis, and apoptosis (1). Excessive glutamate, as a critical pathogenic event, causes brain disorders such as Alzheimer’s disease (AD), and Parkinson’s disease (PD) (2, 3). 

It has been hypothesized that elevated glutamate is mediated by the over-induction of glutamate receptors, leading to increased calcium (Ca^2+^) influx. Another hypothesis is reactive oxygen species (ROS) elevation, depletion of glutathione content, and accumulation of hydrogen peroxide (4). Also, excessive glutamate causes up-regulation of the phosphoinositide-3 kinase/protein kinase B (PI3K/Akt), Akt/mammalian target of rapamycin (mTOR) protein, mitogen-activated protein kinase (MAPK)/extracellular signal-regulated kinase (ERK)1/2, and autophagy cascades that are involved in neurodegenerative diseases pathophysiology, as well (3, 5). 

Plants and plant extracts consist of many components that are additively or even synergistically thought to function on several molecular targets. Nowadays, phytochemicals are applied in neurodegenerative disorders because of their active components that have anti-oxidant properties (6-11). Hence, in the current review, we elaborate on the role of glutamate as a promising target in neurodegenerative diseases and discuss the protective effects of some phytochemicals on glutamate-induced neurotoxicity. 

***Glutamate signaling pathway in neurodegenerative disorders***

Glutamate receptors are classified into two major classes, metabotropic and ionotropic receptors. Ionotropic glutamate receptors (iGluRs) are divided into N-methyl-D aspartic acid (NMDA, high calcium conductivity), kainite (mediate sodium influx), and α-amino-3-hydroxy-5-methyl-4-isoxazole propionic acid (AMPA, mediate sodium influx). In contrast, metabotropic glutamate receptors (mGluRs) are G-protein-coupled receptors (GPCRs) that initiate intracellular cascades, prompting the modification of ion channels (12, 13).

The levels of glutamate are low in the extracellular spaces in the cerebrum because of three major carriers: glutamate transporter 1 (GLT1, expressed in astrocytic cells), excitatory amino acid carrier 1 (EAAC1, manifested into the brain), and glutamate/aspartate transporter (GLAST, expressed in glial cells) (14).

**Figure 1:**
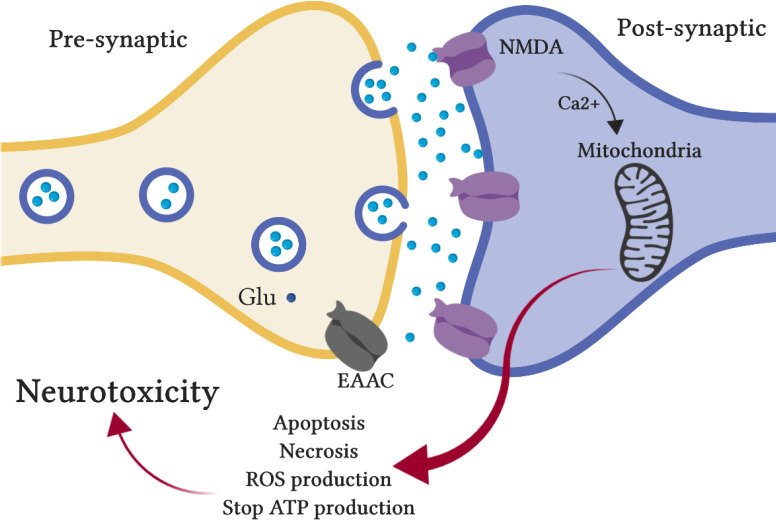
Glutamatergic transmission in the brain. Because of the high release of glutamate from the presynaptic axon, the glutamate receptor (mainly NMDA) is activated, and the raised levels of Ca^2+^ are gathered in the mitochondria. Ca^2+^ overload results in mitochondrial dysfunction. Mitochondrial changes as higher ROS development, adjusted redox potential, and the release of apoptotic mediators, and ATP synthesis failure can promote modified cellular homeostasis causing swelling and cellular disturbance

Neurodegenerative diseases are related to the glutamatergic pathway, prompting the activation of nitric oxide synthase, impairment of cellular calcium homeostasis, ROS generation, and apoptosis. Recently, further mechanistic details identified with glutamate overstimulation have been detailed in neurodegenerative diseases (15). Changes in the enactment pattern of NMDA receptors (NMDARs), as the fundamental receptor involved in neurodegenerative diseases, at various cellular locations have been indicated as crucial in initiating pathways driven to neuroprotection versus neuro-destruction. For this reason, evidence suggests that glutamatergic excitotoxicity is interceded by the activation of extrasynaptic relative to synaptic NMDARs. Various investigations have shown that high enactment of extrasynaptic pathways explicitly promote apoptotic signal transduction cascades, causing neuronal cell death, while the actuation of synaptic NMDARs has a neuroprotective role via Ca^2+^-mediated signal transduction pathways advancing neuronal survival (Figure 1) (16, 17). Mitochondria, as a cytoplasmic organelle and the first modulator of ROS, control the production of ATP, intracellular Ca^2+^, and free-radical scavenging. Neuronal cell death is specifically intervened by the Ca^2+^ entry over NMDARs. Hence, mitochondrial dysfunction, particularly in excitable cells, is in charge of expanding ROS production, and consequently, oxidative damage, promoting neurodegenerative disorders (18, 19).

***Phytochemicals as promising compounds against glutamate-induced excitotoxicity***

 Ethnopharmacological studies have provided data identifying potential new medications taken from plant sources (9). In traditional medicine, various therapeutic plants and natural products have been utilized to treat neurodegenerative disorders (20-22). For instance, many drugs that are accessible in medicine were initially derived from plants, including anti-cholinesterase alkaloids isolated from plants, which have been explored for their likely effects on AD (7, 23). As shown in Table 1, we summarized the potential protective results of specific medicinal herbs against glutamate toxicity.

Different studies have proposed that active compounds isolated from plants conceivably postpone neurodegeneration and improve memory and cognitive function through their anti-inflammatory and anti-oxidant activities (24, 25). Hence, in this section, we reviewed the protective impacts of some of the phytochemicals that have been utilized in glutamate-induced neurotoxicity.

*Astaxanthin*

Astaxanthin (AST), a carotenoid compound, is used as a dietary supplement intended for human, animal, and aquaculture consumption (26). *In vivo* and *in vitro* studies have reported anti-carcinogenic and anti-inflammatory activity, as well as neuroprotective, and powerful anti-oxidative effects of AST (27-29). Research has shown that AST elevated the expression of heme oxygenase-1 (HO-1) and reduced oxidative stress in SH-SY5Y neuroblastoma cells (30). In another study, mouse neuronal cells (HT22) were exposed to different concentrations of AST (1.25–5 µM) and then incubated with glutamate at a dose of 5 mM. The results have shown that AST reduced glutamate toxicity via reduction of cell death, decreased the level of lactate dehydrogenase (LDH), and attenuated the expression of caspase-3/-8/-9 activity, leading to mitochondrial dysfunction by modulating the Akt/glycogen synthase kinase (GSK)-3β signaling pathway in HT22 cells (31). Also, in a different study, the neuroprotective roles and probable mechanisms of AST against glutamate-induced neurotoxicity in PC12 cells were explored. Inhibition of neuronal cell death (reducing the elevation of caspase-3 activation and Bax/Bcl-2 ratio), decreased Ca^2+^ influx, and the down-regulation of ROS-associated NF-κB and MAPK pathways were the main possible mechanisms of AST action (53).

*Berberine*

Berberine, as an isoquinoline alkaloid, is found in various plants, especially in Berberis (54). This natural compound has numerous pharmacological effects (55), such as anti-fungal (56), anti-convulsant (57), anti-tumor (58), anti-viral (59), anti-inflammatory (60), reduction of ischemic brain damage (61), and anti-oxidative activities (62). Also, different studies have reported its neuroprotective effects against neurological diseases, such as anxiety, brain stroke, mental depression, and AD (63). Pharmacokinetic properties have revealed that berberine rapidly passes through the blood brain barrier, while slowly eliminating (64). Sadeghnia *et al.* investigated the protective effect of berberine (50–1000 µM) against glutamate excitotoxicity in PC12 and N2a cells. It was found that berberine reduced ROS, malondialdehyde (MDA), DNA fragmentation, and increased superoxide dismutase (SOD) activity. Also, berberine attenuated the level of caspase-3 and Bax/Bcl-2 ratio (65). In agreement with their results, Lin *et al.* have shown that berberine prevented glutamate release from rat’s cortical synaptosomes by the inhibition of presynaptic Cav2.1 channels and the ERK/synapsin I signaling pathway (66). Furthermore, a recent study has shown that berberine (25 μg/ml) improved viability, axonal outgrowth, and transport in calyculin A-injured N2a cells. The neuroprotective effect of this compound was related to anti-oxidant activity, decreasing of MDA, increasing of SOD, and inhibiting the hyper-phosphorylation of Tau protein, and neurofilaments (67). Besides, berberine possesses neuroprotective effects through anti-oxidant defense stimulation, oxygen consumption inhibition, and mitochondrial membrane potential (MMP) elevation (68). Preclinical studies have demonstrated that berberine has shown important memory improvement activities with several mechanisms, including anti-inflammatory, anti-amyloid, anti-oxidant, and cholinesterase inhibitory activities (69). Based on these findings, berberine could be a useful agent to improve AD and PD by modulating oxidative stress (70). 

*Casuarinin*

Casuarinin, an ellagitannin, exists in the pericarp of pomegranates (*Punica granatum*). In a study by Song *et al.*, casuarinin reduced glutamate-induced HT22 murine hippocampal neuronal cell death by inhibiting ROS production, reducing chromatin condensation, and inhibiting oxidative stress-mediated MAPK phosphorylation (71).

*Chebulinic acid*

Chebulinic acid isolated from the extracts of the *Terminalia chebula *attenuates glutamate-induced HT22 cell death by inhibiting calcium influx, oxidative stress, and MAPKs phosphorylation (72).

*Cinnamaldehyde*

Cinnamaldehyde is an important component of cinnamon oil that is obtained from the stem bark of *Cinnamomum cassia* (73). It has various pharmacological properties, such as anti-oxidant (74), anti-bacterial (75), anti-inflammatory (76), and anti-tumor (77) activities. Also, cinnamaldehyde, as a potential modulator of dopaminergic neurons in the substantia nigra, has beneficial effects in neurotoxin-induced disorders, including PD (78).

Suppression of NMDA receptors may be relevant to cinnamaldehyde neuroprotective effects against amyloid-β-induced neurotoxicity (79). In a study, the protective effects of cinnamaldehyde (5, 10, 20 μM) on glutamate-induced excitotoxicity were examined in PC12 cells. Glutamate at a concentration of 4 mM caused the generation of ROS and reduced the level of glutathione (GSH) and SOD activity. Pretreatment of the cells with cinnamaldehyde reduced cell death, ROS production, the activity of caspase -3/-9, and the release of cytochrome C, and modulated the expression of the Bcl-2 family (80).

*Curcumin*

Over the past ten years, the impact of curcumin on various diseases was reported to have hepatoprotective, cardioprotective, anti-carcinogenic, thrombo-suppressive, anti-arthritis, and anti-infective activities (81). Lately, curcumin, as a significant constituent of *Curcuma longa*, has been reported to have anti-excitotoxicity activity, as well (82). A major enthusiasm has been created due mainly to the lack of toxicity and low cost of curcumin in several preclinical trials for neurodegenerative diseases (83). Several studies have demonstrated that curcumin counteracts the depolarization-evoked release of glutamate by reducing voltage-dependent Ca^2+^ entering from nerve terminals in rat (84). Also, curcumin protects neurons from excitotoxicity caused by glutamate through the AKAP79-PKA membrane-anchored interaction network and decreasing mGluR5 and NMDA expression (85, 86). Wang *et al.* have found that curcumin protects the cerebral cortical neurons of rats against glutamate excitotoxicity by increasing brain-derived neurotrophic factor (BDNF) level and activating tropomyosin receptor kinase B (TrkB) (87). Chang *et al.* have reported that curcumin has protective effects in PC12 cells against the glutamate-induced neurotoxicity through glutathione-dependent nitric oxide- ROS pathway and the mitochondria-dependent nitric oxide- ROS pathway (88). Furthermore, Suh *et al.* have shown that by blocking MAPK signals, curcumin alleviates glutamate-induced HT22 cell death (89). The results of an encouraging study have shown that curcumin attenuated the glutamate-induced neurotoxicity by inhibiting endoplasmic reticulum stress-associated TXNIP/NLRP3 inflammasome activation in a manner dependent on 5’ AMP-activated protein kinase (90). Hence, curcumin could be a therapeutic agent in the treatment of neurotoxic situations.

*Ginkgolide K*

Ginkgolide K, as an active compound isolated from leaves of the *ginkgo Biloba*, inhibits the release of beta-glucuronides from platelet (a platelet-activating factor antagonist) and ameliorates neurotoxicity induced by cerebral ischemia (91, 92). Various doses of ginkgolide K (10, 50, 100 mM) were added to PC12 cells with glutamate (10 mM) for 12 hrs. Glutamate has been shown to increase the concentration of Ca^2+^ influx, MDA, Bax/Bcl-2 ratio, LDH, caspase-3 protein, the release of cytochrome C, and ROS production. Also, it leads to a decrease in SOD, cell viability, MMP, and GSH peroxidase activity. The outcomes revealed that ginkgolide K attenuated the toxicity of glutamate in PC12 cells by various mechanisms, such as preventing Ca^2+^ influx, ROS production, and apoptosis reduction (93).

*Huperzine A*

Huperzine A, a naturally occurring sesquiterpene alkaloid found in *Huperzia serrata*, has been investigated as a promising treatment for neurological conditions such as AD. The protective effects of huperzine A against oxidative glutamate toxicity in mouse-derived hippocampal HT22 cells were evaluated to explore its probable mechanisms. It was shown that huperzine A attenuated oxidative glutamate excitotoxicity in murine hippocampal HT22 cells via activating the BDNF/TrkB-dependent PI3K/Akt/mTOR signaling pathway (94). 

*Paeoniflorin*

The primary active component of the aqueous extract of *Radix Paeoniae alba* is paeoniflorin (PF). This ingredient, as a monoterpene glycoside, can enhance memory and learning and has anti-oxidant and sedative properties (95, 96). It has been revealed that PF (0.1, 1.0, and 10 μM) reduce glutamate toxicity in PC12 cells, in a dose-dependent manner. Its protective effect was attributed to the inhibition of apoptosis via regulation of Bax/Bcl-2 signaling and MMP (97). In agreement with this finding, Mao *et al.* hypothesized that the possible protective mechanisms of PF are through anti-oxidant mechanisms and Ca^2+^ antagonism in PC12 cells (98). Besides, pretreatment of PC12 cells with PF (100, 200, and 300 μM) inhibited the cytotoxicity of glutamate (15 mM) by increasing Bcl-2, Bcl-xL, reducing Bax and Bad expression, and preventing caspase-3/-9 activity (99). So, PF seems to be a potential natural compound for the treatment of neurodegenerative disorders.

*Naringenin*

Naringenin, as an anti-oxidant bioflavonoid isolated from *Dracocephalum rupestre*, is found in a variety of fruits and herbs (100). In a study, the neuroprotective impacts of naringenin on excitotoxicity incited by glutamate in primary hippocampal neurons of neonatal mice were investigated. Naringenin directed ERK1/2 and Akt phosphorylation and decreased the degeneration of dendrites because of glutamate exposure in cultured hippocampal neurons. Besides, naringenin actuated the BDNF and other neuroprotective cytokines and notably improved the survival rates of the neurons 24 hrs following glutamate exposure (101).

*Protopanaxadiol*

Protopanaxadiol (PPD), characterizing a group of ginsenosides, is found in ginseng (*Panax ginseng*) and notoginseng (*Panax pseudo ginseng*). Bak *et al*. demonstrated the neuroprotective effects of 20(S)-PPD (10 µM) against excitotoxicity induced by glutamate (5 mM) in PC12 cells. PPD has been shown to prevent glutamate-induced apoptosis via improvement of mitochondrial function and anti-oxidant activity (102).

*Tanshinone*

Tanshinone, isolated from *Salvia miltiorrhiza*, has been proposed to have cytotoxicity as well as anti-inflammatory and anti-oxidative effects on a variety of cells and modulates breast cancer metastasis by regulating adhesion molecules (103, 104). Tanshinone is divided into three classes: dihydrotanshinone, tanshinone I, or tanshinone IIA. In Li *et al. *study, it was shown that tanshinone IIA mitigates glutamate-induced oxidative toxicity through the suppression of mitochondrial dysfunction and inhibition of MAPK activation in neuroblastoma cells (105).

*Thymoquinone*

Thymoquinone, as volatile oil, is abundantly found in *Nigella sativa* (10). It has different therapeutic effects, such as anti-oxidant, anti-cancer, anti-inflammatory, and neuroprotective actions (106-111). Also, the neuroprotective effect of thymoquinone has been demonstrated against neurotoxic agents such as amyloid-β and α-synuclein peptides (109, 111). The SH-SY5Y cells were exposed to different concentrations of thymoquinone (0.1–3 µM) for 18 hours, and then glutamate (8 mM) was added for 8 hours. Findings have shown that glutamate increased cell death, ROS production, and disturbance of the mitochondrial and apoptosis pathway by increasing caspase-9 and Bax expression, and reducing Bcl-2, while thymoquinone attenuated glutamate toxicity via reduction of ROS generation and apoptosis (112). Besides, in a placebo-controlled clinical trial, *Nigella sativa* seed capsule (500 mg, twice daily for nine weeks) was shown to improve cognition, memory, and attention in elderly volunteers (113). Hence, *Nigella sativa*, especially its constituent, Thymoquinone, could be a promising neuromodulator agent for the treatment of neurodegenerative diseases.

The pharmacological mechanisms of the discussed- phytochemicals against glutamate-excitotoxicity is demonstrated in Table 2.

Glu: Glutamate; NMDA: N-Methyl-d-aspartic acid; EAAC: Excitatory amino acid carrier; ROS: Reactive oxygen species

**Table 1:** The protective effects of some of medicinal plants on glutamate-induced neurotoxicity

**Medicinal plant**	**Family**	**Type of study**	**Protocol**	**Findings**	**Reference (s)**
***Acanthus ebracteatus ***	Acanthaceae	*In vitro*	Treatment of HT22 cells with different concentrations of extract (3-50 µg/ml) and glutamate ‎(5 mM) for 24 hours	1) Reduction of cell death via attenuation of ROS generation, nuclear AIF translocation 2) ‎Activation of the Nrf2/ARE‎ pathway	(24)
***Alpinia oxyphylla***	*‎*Zingiberaceae	*In vitro*	Co-treatment of primary cultured mouse cortical neurons with the extract (80-240 µg/ml) and glutamate (30 µM)	1) An increase in the cell viability 2) A reduction in the number of apoptotic cells 3) Decrease in the intensity of DNA fragmentation.	(25)
***Amburana cearensis ***	Fabaceae	*In vitro*	Pretreatment of PC12 cells with glutamate at a dose of 1 mM, then exposed to different doses of the extract ‎ ‎(0.1-1000 µg/ml)	Anti-oxidant activity ‎	(26)
***Aronia melanocarpa***	Rosaceae	*In vitro*	Co-treatment of HT22 cells with the extract at doses of 10 and 100 µg/ml and glutamate (2 mM) for 24 hours	1) Reduction in ROS level 2) A decrease in intracellular Ca^2+^ 3) Increase in anti-oxidant enzymes	(27)
***Boswellia serrata***	Burseraceae	*In vitro*	Co-treatment of N2a and PC12 cells with the extract (25-100 μg/ml) and glutamate (8 mM)	Amelioration of the oxidative stress and the resultant apoptosis	(28)
***Calendula officinalis***	Asteraceae	*In vivo*	The rats received oral extracts at doses of 100 and 200 mg/kg, 1 hour after monosodium glutamate injection for seven days.	Improvement in oxidative stress, hippocampal damage, and behavioral changes	(29)
***Citrus aurantium***	Rutaceae	*In vitro*	Pretreatment of PC12 cells with the extract alone (6 to 200 µg/ml) for 2 hours and then incubation with glutamate (8 mM) for 24 hours	1) Reduction in ROS level 2) Reduction in MDA level 3) Reduction in apoptotic cells	(30)
***Cymbopogon citratus and Ferula assafoetida***	Poaceae and Apiaceae	*In vitro*	Cerebellar granule neurons were treated with extract at a dose of 100 μg/ml before, after, and during exposure to 30 μM of glutamate.	Reduction in cell death and apoptosis	(31)
***Ferula gummosa***	Apiaceae	*In vitro*	Pretreatment of PC12 and N2a cells with different concentrations of extract (25 to 200 μg/ml) for 2 hours, then incubation with glutamate (8 mM) for 24 hours	1) Reduction in ROS level 2) Reduction in MDA level 3) Reduction in apoptotic cells	(32)
***GLGZD*** ***‎*** *** (extracts of Zingiber officinale, Trichosanthis Radix, Paeonia lactiflora, Ramulus Cinnamomi, Fructus Jujubae, Roscoe and Glycyrrhiza)***	*-*	*In vitro*	Co-treatment of BV-2 microglial cells with different concentrations of extract (125 to 1000 μg/ml) and glutamate (30 mM) for 24 hours	1) Down-regulation of Bax/Bcl-2 ratio 2) Inhibition of caspase-3 expression	(33)
***Glycine max (soybean)***	Fabaceae	*In vitro*	Incubation of cortical cell cultures with the indicated compound for one hour and then exposed to 100 μM glutamate for 24 hours	Inhibition of glutamate-induced toxicity via the neuroprotective effects of triterpene glycosides	(34)
***Polygonum multiflorum Thunb***	Polygonaceae	*In vitro*	Pretreatment of HT22 cells with various concentrations of the extract (0.1 to 10 µg/ml) for 24 hours and then exposed to glutamate (5 mM) for 24 hours	1) Inhibition of glutamate-induced oxidative neuronal death 2) Prevention of ERK and p38 activation	(35)
***Reseda luteola***	Resedaceae	*In vitro*	Co-treatment of HT22 cells with 20 μg/ml of the extract and 5 mM of glutamate for 24 hours	Enhancement of anti-oxidant systems, such as HO-1, peroxiredoxin, catalase, and NQO1 gene	(36)
***Rheum turkestanicum *** ***‎***	Polygonaceae	*In vitro*	Pretreatment of PC12 and N2a cells with various concentrations of the extract (6-‎‎200 µg/ml), then incubation with glutamate	Reduction in cell death, apoptosis, lipid peroxidation, and ROS generation‎	(37)
***Rhinacanthus nasutus***	Acanthaceae	*In vitro*	Treatment of HT-22 cells with extract (0.1, 1 and 10 µg/ml) and glutamate (5 mM) for 18 hours	Anti-oxidant activities	(38)
***Saussurea pulvinata Maximo***	Compositae	*In vitro*	Pretreatment of PC12 cells with different concentration of the extract (10^−7^, 3×10^−7^, or 10^−6^ M) for 1 hour, and then exposing to glutamate (5 mM) for 24 hours	1) Anti-oxidative effects 2) Anti-apoptotic properties	(39)
***Scrophularia genus***	Scrophulariaceae	*In vitro*	Exposing cerebellar granule neurons to 125 μM of glutamate for 12 hours following 24 hours of incubation with extract (10 mcg/ml)	Reduction of oxidative stress	(40)
***Solanum torvum***	Solanaceae	*In vivo*	Mice received the oral extract (100 and 300 mg/kg) at doses of monosodium glutamate (1000 mg/kg) for 14 days	Improvement in behavioral tests decreased lipid-peroxidation, while increased anti-oxidant content ‎such as catalase, SOD and glutathione‎	(41)
***Uncaria sinensis***	Rubiaceae	*In vitro*	1) Pretreatment of primary cultured cortical neurons with extract (1 and 5 µg/ml), then exposing to glutamate (200 μM) 2) Incubation of cerebellar granule cells with different concentrations (3 to 300 µM) of testing materials with glutamate (100 µM)	1) Inhibition of death receptor 4 2) Expression of anti-apoptotic proteins XIAP and Bcl-2 3) Inhibition of Ca^2+^ influx	(42, 43)
***Withania somnifera***	Solanaceae	*In vitro*	Pretreatment of rat glioma (C6) and human neuroblastoma (IMR-32) cells with the extract (0.05% and 0.1%), then exposing to glutamate (0.06 mM-10 mM)	Inhibition of glutamate-induced cell death	(44)

ROS: Reactive oxygen species; AIF: Apoptosis-inducing factor; Nrf2: Nuclear factor erythroid 2-related factor 2; ARE: Anti-oxidant responsive element; MDA: Malondialdehyde; ERK: Extracellular-signal-regulated kinase; HO-1: Heme oxygenase-1; SOD: Superoxide dismutase; XIAP: X-linked inhibitor of apoptosis

**Table 2:** Structure and pharmacological properties of specific phytochemicals against glutamate-induced neurotoxicity

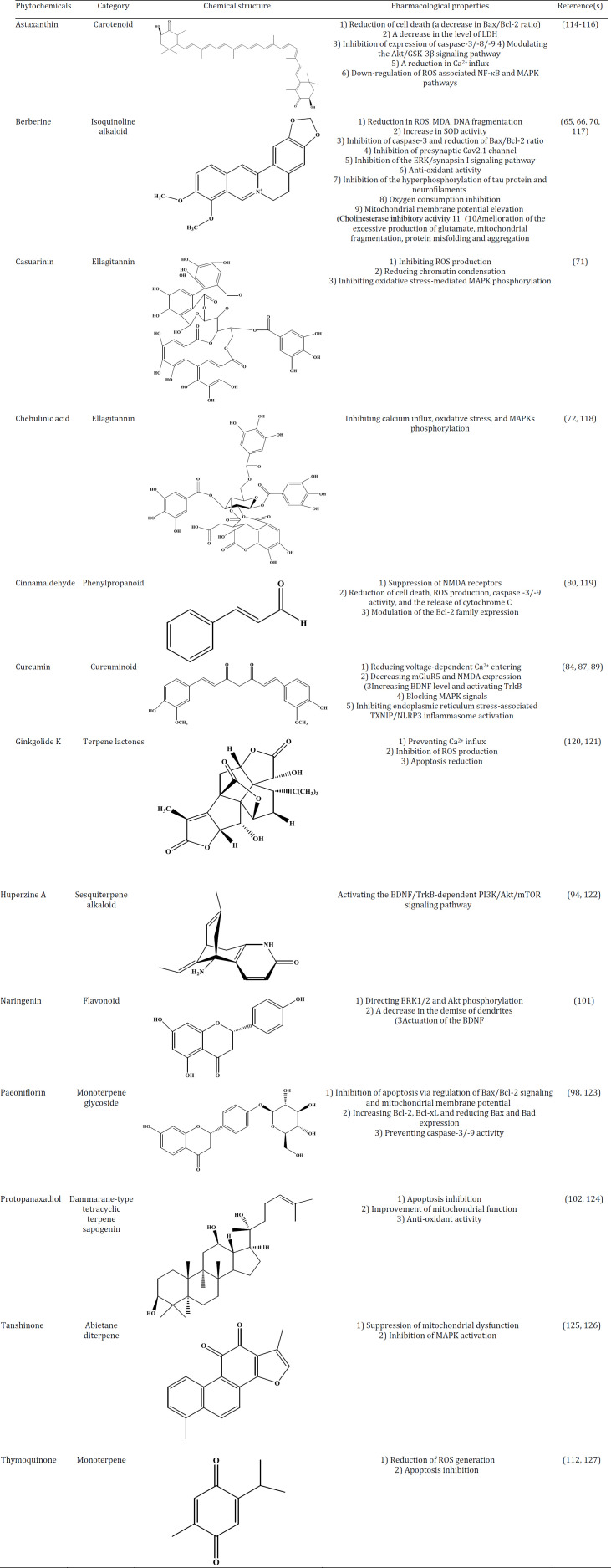

ROS: Reactive oxygen species; MDA: Malondialdehyde; SOD: Superoxide dismutase; ERK: Extracellular signal-regulated kinase; LDH: Lactate dehydrogenase; Akt: Protein kinase B; GSK-3β: Glycogen synthase kinase-3 beta; MAPK: Mitogen-activated protein kinase; NF-κB: Nuclear factor-kappa B; NMDA: N-methyl-D-aspartic acid; mGluR: Metabotropic glutamate receptor; BDNF: Brain-derived neurotrophic factor; ‎:TrkB Tropomyosin receptor kinase B; PI3K: Phosphoinositide 3-kinase; mTOR: Mammalian target of rapamycin

## Conclusion

The idea of excitotoxicity as a vital mechanism to comprehend neurotoxicity has made significant progress in determining its role in the pathogenesis of neurodegenerative disorders. It is currently accepted that glutamate is the major excitatory neurotransmitter, and when its level elevates over its threshold value, it leads to excitotoxicity. In this review, we concentrated on different induced neurotoxicities in various studies (*in vitro* and *in vivo*) and investigated the protective effects of plants and their constituents on induced toxicity in neural cells. Because of the limited studies available that evaluate their neuroprotective activities, neuroprotective properties of natural compounds, especially curcumin, cinnamaldehyde, thymoquinone, and astaxanthin, need to be discussed more. Most of these promising phytochemicals could play their neuroprotective roles through a reduction in neuronal cell death, restoration of GSH, a decrease in MDA levels, preventing caspases activity, modulation of oxidative stress and protection of neurons against ROS as anti-oxidant activities, reduction of Ca^2+^, and anti-inflammatory activities. Hence, our research strongly indicates the potential for the use of phytochemicals as pharmacological targets in neurotoxic situations in the future. 
